# Fault Detection and Isolation of MEMS IMU Array Based on WOA-MVMD-GLT

**DOI:** 10.3390/mi17030374

**Published:** 2026-03-19

**Authors:** Hanyan Li, Fayou Sun, Jingbei Tian, Xiaoyang He, Ting Zhu

**Affiliations:** 1 School of Automation, Guangxi University of Science and Technology, Liuzhou 545006, China; lihanyan@gxust.edu.cn (H.L.); 20230203013@stdmail.gxust.edu.cn (F.S.); 2005090@gxust.edu.cn (J.T.); 20240202048@stdmail.gxust.edu.cn (X.H.); 2 Guangxi Key Laboratory of Logistics Unmanned Aircraft Technology for Transportation Industry, Liuzhou 545616, China; 3 Guangxi Low-Altitude Unmanned Aircraft Key Technologies Engineering Research Center, Liuzhou 545616, China

**Keywords:** MEMS IMU array, fault detection and isolation, whale optimization algorithm, multivariate variational mode decomposition, generalized likelihood test

## Abstract

The stable and accurate output of the inertial measurement unit array (IMU) of a micro-electro-mechanical system (MEMS) is the key to ensuring the data fusion of the MEMS IMU array. However, due to the large number of MEMS IMUs contained in the MEMS IMU array, it is susceptible to interference and has difficulty avoiding failures. The output of the MEMS IMU contains noise, outliers, and other related errors, which can seriously lead to low fault detection and isolation accuracy in the MEMS IMU. In this study, a new method of fault detection and isolation based on multivariate variational mode decomposition (MVMD), a whale optimization algorithm (WOA), and a generalized likelihood test (GLT) is proposed, which is called WOA-MVMD-GLT. Firstly, a multi-index fitness function WOA is proposed to optimize the parameters of MVMD. Secondly, MVMD is used to extract the features of the MEMS IMU’s signals. Finally, a GLT is used to construct a fault detection function and a fault isolation function to detect and isolate the faults of gyroscopes and accelerometers. The experimental results show that the method proposed in this paper can significantly reduce the false alarm rate and false isolation rate.

## 1. Introduction

The MEMS IMU array is constructed from multiple low-cost MEMS IMUs in an array manner, and its accuracy is improved through data fusion to achieve low-cost and high-precision inertial measurement. The MEMS IMU array, as the core of an inertial navigation system, provides the angular velocity and acceleration information required by the carrier and is a key component for achieving highly reliable navigation. In 2003, Bayard et al. first proposed MEMS IMU array technology [1]. Bayard took a MEMS gyroscope as the research object to form a gyroscope array, and its performance has been significantly improved since then. In 2008, Tanenhaus et al. developed an IMU array that consisted of 24 MEMS gyroscopes and six accelerometers [2]. Wang et al. successfully assembled 72 MEMS gyroscopes using a three-layer PCB hardware design in a University of Michigan laboratory in 2015 [3]. In 2019, Owais et al. used an FPGA to design data acquisition hardware for a MEMS IMU array and performed fusion experiments on the collected array data, indicating that the research on MEMS IMU arrays was entering the application stage [4,5]. MEMS IMU arrays have been used in aerospace, unmanned driving, robotics, medical equipment, and other applications since 2024 [6,7]. However, due to the large number of sensors, it is difficult to avoid failures. When some inertial sensors fail, it can disrupt the fusion of MEMS IMU array data, and the unmerged information may contaminate all information on navigation, posing serious safety risks and damaging the performance of the inertial navigation system. Therefore, it is necessary to perform fault detection and isolation on MEMS IMU arrays.

At present, there are few research methods for the fault detection and isolation of MEMS IMU arrays. We draw on redundant IMUs and other related methods in this research. Many researchers have adopted the parity space approach for the fault detection and isolation of redundant IMUs, mainly utilizing the generalized likelihood test method (GLT) [8,9], the optimal parity vector method [10,11,12], and the singular value decomposition method [13], which differ mainly in their decoupling matrix. Later, researchers’ work mainly focused on improving the parity space approach in order to enhance the navigation performance of the redundant IMU. For example, Kwang [14] and Lee [15] proposed a new method applicable to two faulty sensors. Wen [16] improved the parity vector method and achieved a lower false alarm rate. Many researchers also use Kalman state estimation values to compare with the estimation values of a shadow filter to form residuals for fault diagnosis. Zou et al. [17] proposed a fault diagnosis and fault-tolerant compensation (FDFTC) strategy for the wheel angle sensor of the SBW vehicle via an extended Kalman filter (EKF). Han et al. [18] proposed a new method for unmanned aerial vehicle fault detection based on quadratic Kalman filtering (QKF), which can accurately detect sudden and early faults of IMU. Mounier et al. [19] combined the optimal dual objective function of the differential evolution (DE) algorithm for fault detection parameter optimization. However, the Kalman filter will absorb faults and the fault amplitude will continuously decay, which is not conducive to the detection of soft faults. In recent years, methods based on neural networks have been increasing. A neural network framework was applied to fault detection [20,21]. Chen et al. [22] proposed a fault detection method based on an enhanced GRU without isolating the faults. In 2024, Dominik Łuczak [23] proposed a promising and efficient approach for vibration classification using IMU data with the proposed IMU6DoF-SST-CNN method. In 2025, Song et al. [24] proposed a hybrid model of CNNs and a Transformer encoder architecture for IMU mutations and early failures. In short, there are many methods based on neural networks, but they need a large amount of sample data, and the MEMS IMU array samples are small, which is not suitable for the field investigated in this study. So far, no researchers have proposed a comprehensive method for fault detection and isolation of the MEMS IMU array. In recent years, intelligent optimization algorithms have continued to emerge, making it possible to provide methods that combine signal processing. Among them, multivariate variational mode decomposition (MVMD) [25] is a multi-channel signal feature extraction algorithm that has significant advantages in processing multi-channel signals output by MEMS IMU array. However, the key parameters of the MVMD algorithm, such as the penalty coefficient and the number of decomposed modes, have a significant impact on the signal decomposition effect. The key parameters usually depend on the selection of manual experience, and the whale optimization algorithm (WOA) [26] can adaptively find the optimal parameter value.

In this paper, a new method of fault detection and isolation based on MVMD-GLT is proposed. The remainder of this paper is structured as follows: Section 2 provides the theory of the WOA-MVMD-GLT method. Section 3 provides experimental results and analysis of fault detection and isolation. Section 4 presents the conclusions of this paper.

## 2. Method

### 2.1. Building Fault Detection and Isolation Functions

**Figure 1:**
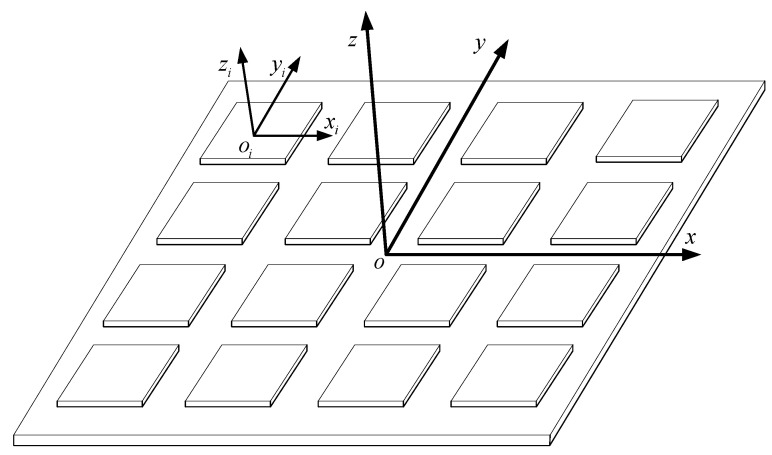
The structure of the MEMS IMU array.

Assuming there are *m* IMUs in the MEMS IMU array, the total number of gyroscopes and accelerometers is the same, with a total of 3*m*. We define the output measurement equation of a gyroscope or accelerometer that only includes noise interference as follows:

(1) $$u=Hx+\varepsilon +b_{f}$$

where u represents the output measurement value of the gyroscope or accelerometer. x represents the true value of the gyroscope or accelerometer. $b_{f}$ represents the fault vector. ε represents the noise vector. H represents the MEMS IMU array configuration matrix. The structure of the MEMS IMU array is shown in Figure 1, and the configuration matrix is as follows:

(2) $$H={\left[\begin{array}{ccc} 1 & 0 & 0\\ 0 & 1 & 0\\ 0 & 0 & 1\\ . & . & .\\ . & . & .\\ . & . & .\\ 1 & 0 & 0\\ 0 & 1 & 0\\ 0 & 0 & 1 \end{array}\right]}_{3m\times 3}$$

Assuming the noise is zero-mean Gaussian white noise, it is satisfied as follows:

(3) $$\left\{\begin{array}{l} E(\varepsilon )=\;0\\ E(\varepsilon \varepsilon^{T})=\sigma^{2}I_{3m} \end{array}\right.$$

We define the parity vector as follows:

(4) $$P_{v}=Vu$$

where $P_{v}$ is the parity vector; V is a parity matrix, which is an unknown matrix. Substitute Equation (1) into Equation (4) as follows:

(5) $$P_{v}=VHx+V\varepsilon +Vb_{f}$$

From the above, it is critical to calculate the matrix V before calculating the parity vector $P_{v}$. We can compute the parity matrix V by using Potter’s algorithm [27].

The parity vector $P_{v}$ shows inconsistent properties in the presence and absence of faults, which can be used as the basis for fault detection. We assume that the inertial sensor has two states, the fault-free state $A_{0}$ and the faulty state $A_{1}$. The statistical characteristics of the two states are as follows:

(6) $$\left\{\begin{array}{l} A_{0}:E(P_{v})=\;0,E(P_{v}{P_{v}}^{T})=\sigma^{2}VV^{T}\\ A_{1}:E(P_{v})=\;\mu ,E((P_{v}-a){{(P}_{v}-a)}^{T})=\sigma^{2}VV^{T},a=Vb_{f} \end{array}\right.$$

The likelihood functions corresponding to the two states are as follows:

(7) $$\left\{\begin{array}{l} \varphi (P_{v}/A_{0})=C\exp\left\{-\frac{1}{2\sigma^{2}}{P_{v}}^{T}{\left(VV^{T}\right)}^{-1}P_{v}\right\}\\ \varphi (P_{v}/A_{1})=C\exp\left\{-\frac{1}{2\sigma^{2}}{\left(P_{v}-a\right)}^{T}{\left(VV^{T}\right)}^{-1}(P_{v}-a)\right\} \end{array}\right.$$

where C represents a constant. σ represents the standard deviation. Construct the logarithmic likelihood ratio function as follows:

(8) $$\begin{array}{l} L(P_{v})=\ln\frac{\varphi (P_{v}/A_{1})}{\varphi (P_{v}/A_{0})}\\ =\frac{1}{2\sigma^{2}}\left[\left({P_{v}}^{T}{\left(VV^{T}\right)}^{-1}P_{v})-({\left(P_{v}-a\right)}^{T}{\left(VV^{T}\right)}^{-1}(P_{v}-a)\right)\right] \end{array}$$

Derive a from the above equation to obtain the maximum likelihood estimate as $\hat{a}=P_{v}$. Substitute the maximum likelihood estimation into (8) as follows:

(9) $$L(P_{v})=\frac{1}{2\sigma^{2}}\left[\left({P_{v}}^{T}{\left(VV^{T}\right)}^{-1}P_{v}\right)\right]$$

The fault detection function is defined as follows:

(10) $$DF_{D}=\frac{1}{\sigma^{2}}\left[\left({P_{v}}^{T}{\left(VV^{T}\right)}^{-1}P_{v}\right)\right]$$

If $DF_{D}>T_{D}$, we define that there are failures.

If $DF_{D}\leq T_{D}$, we define that there are no failures.

Here, $T_{D}$ represents the threshold. If we take the significance level as 0.01, then $T_{D}$ can be obtained from $\chi^{2}$ the distribution table with degrees of freedom 3m−3.

After detecting the fault, it is also necessary to isolate the faulty sensor to prevent damage to the inertial measurement system. The assumptions are as follows:

C: The *i*-th sensor has failed, i=1,2,3,⋯,3m.

The fault vector can be rewritten as follows:

(11) $$b_{f}=e_{i}f$$

where $e_{i}$ represents the unit vector. f represents the size of the fault vector. The results are as follows:

(12) $$\mu =Vb_{f}=Ve_{i}f=fv_{i}$$

where $v_{i}$ represents the *i*-th column vector of V.

The corresponding statistical characteristics are as follows:

(13) $$C_{i}:E\{P_{v}\}=fv_{i},E\{(P_{v}-fv_{i}){\left(P_{v}-fv_{i}\right)}^{T}\}=\sigma^{2}VV^{T}$$

The likelihood function is as follows:

(14) $$\varphi (P_{v}/C_{i})=K\exp\left\{-\frac{1}{2\sigma^{2}}{\left(P_{v}-fv_{i}\right)}^{T}{\left(VV^{T}\right)}^{-1}(P_{v}-fv_{i})\right\}$$

The log likelihood function is constructed as follows:

(15) $$L(P_{v})=\ln\varphi (P_{v}/C_{i})=\ln K-\frac{1}{2\sigma^{2}}({\left(P_{v}-fv_{i}\right)}^{T}{\left(VV^{T}\right)}^{-1}(P_{v}-fv_{i}))$$

The maximum likelihood estimate derived from the above equation is as follows:

(16) $$\hat{f}=\frac{{P_{v}}^{T}{\left(VV^{T}\right)}^{-1}v_{i}}{{v_{i}}^{T}{\left(VV^{T}\right)}^{-1}v_{i}}$$

The fault isolation function by substituting the maximum likelihood estimation into Equation (15) is as follows:

(17) $$DFI_{i}=\frac{1}{\sigma^{2}}\frac{{\left[\left({P_{v}}^{T}{\left(VV^{T}\right)}^{-1}v_{i}\right)\right]}^{2}}{{v_{i}}^{T}{\left(VV^{T}\right)}^{-1}v_{i}}$$

The sensor corresponding to the maximum fault isolation function is the fault sensor.

### 2.2. Analyzing the Impact of Errors on Fault Detection

In practical engineering applications, IMU output includes installation errors, scale factor errors, outliers, and other random errors, which will change with the maneuvering of the carrier and affect the effectiveness of GLT fault detection. In order to reduce the impact of the random error, one method is to set an adaptive threshold; that is, the carrier sets different threshold values under different motion conditions, but the threshold is usually set as relatively large, resulting in a decline in the system’s fault detection ability. The other is to use the filtering method or the deviation separation estimation method to compensate for the odd and even vectors, so that the odd and even vectors are not sensitive to the error state, and finally realize the error compensation effect. But these methods need a more accurate dynamic model and noise statistical characteristics, which are not easy to obtain in practice.

Considering that the output of the inertial sensor contains error terms such as installation error, scale coefficient error, and outliers, (1) is rewritten as follows:

(18) $$u=(I+A_{se})\left(H+H_{e}\right)x+\varepsilon +b_{f}$$

where $A_{se}\in R^{3m\times 3m}$ represents the scale coefficient error matrix. $H_{e}\in R^{3m\times 3}$ represents the installation error matrix.

Since the order of magnitude of installation error and scale factor error is small and negligible, this paper only studies random error, $A_{se}\approx 0,H_{e}\approx 0$. $P_{v}$ is expressed as follows:

(19) $$\left\{\begin{array}{l} P_{v}=VHx+V\varepsilon ,no\;fault\\ P_{v}=VHx+V\varepsilon +Vb_{f},fault \end{array}\right.$$

**Figure 2:**
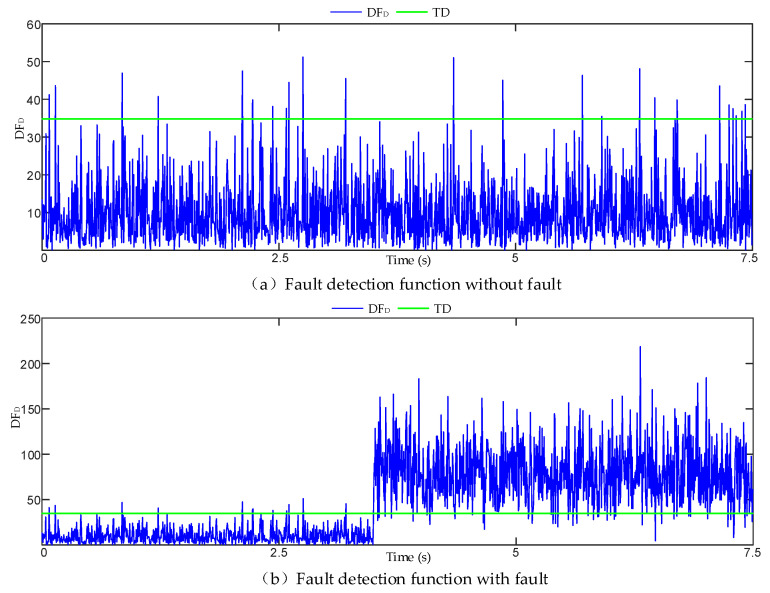
Comparison of fault detection functions. (**a**) Fault detection function curve when no fault occurs. (**b**) Fault detection function curve when a fault occurs.

**Figure 3:**
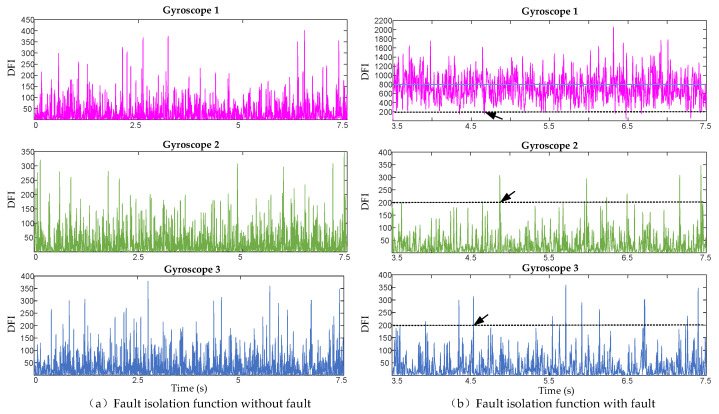
Comparison of fault isolation functions. (**a**) Fault isolation function curve when no fault occurs. (**b**) Fault isolation function curve when a fault occurs. Arrows indicate false alarms.

It can be seen from the above that fault detection and isolation are sensitive to random error terms. To verify the impact of random error terms on fault detection and isolation, we simulated the array data composed of six IMUs and added step faults to the No. 1 gyroscope at 3.5 s. The fault detection and isolation curves are shown in Figure 2 and Figure 3, respectively. Figure 2 shows that when no fault occurs, more faults are still detected. After 3.5 s, the value of the fault detection function increased significantly, and there was a situation of fault missing detection due to the interference of random error. Figure 3 shows that after the failure of 3.5 s, the average amplitude of fault isolation of No. 1 gyroscope is higher than that of other gyroscopes, but there are many false isolation phenomena similar to those indicated by the arrow in the figure, which are caused by the interference of random error.

When the error is not compensated, $P_{v}$ is mainly affected by the random error term, which will make the fault detection and isolation function deviate and reduce the accuracy. In order to improve the performance of fault detection and isolation, it is important to estimate the inaccuracies of all sensors as accurately as possible to obtain more accurate measurement equations, so that these errors will not affect the calibration equation. However, not all errors are observable. Using the MVMD algorithm for feature extraction can suppress the interference of the random error term of the array data.

### 2.3. Feature Extraction of the Signal

**Figure 4:**
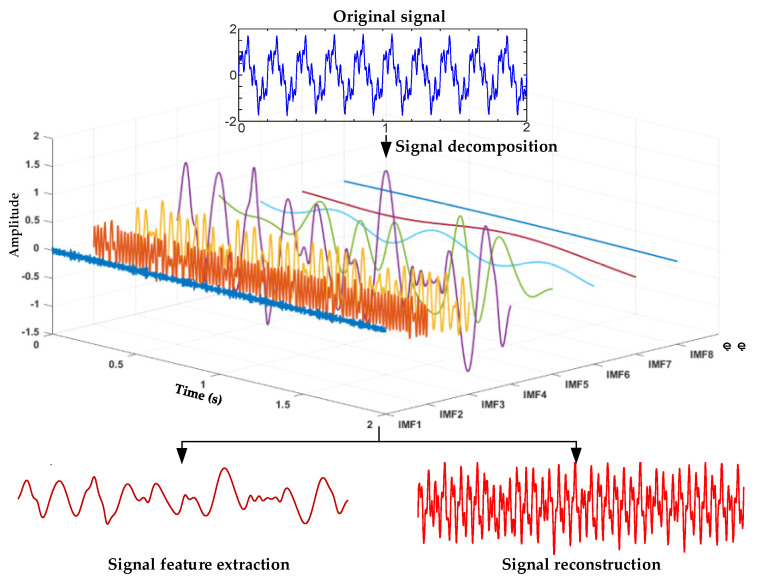
Schematic diagram of MVMD.

MVMD is an extension of the variational mode decomposition (VMD) [28], mainly used for processing complex and multi-channel signals. The core idea is to decompose the collected original signal into several modal components with different frequencies, which can be analyzed, and extract different features of the signal. The main process of MVMD is shown in Figure 4.

All gyroscope or accelerometer data collected by the MEMS IMU array are used as input data for MVMD, and the input data are represented as follows:

(20) $$d(t)=[d_{1}(t),d_{2}(t),\ldots ,d_{C}(t)],C=3m$$

MVMD decomposes d(t) into K modal components as follows:

(21) $$d(t)=\sum_{k=1}^{K}u_{k}(t)$$

where $u_{k}(t)$ represents the *k*-th multivariate oscillations obtained by MVMD.

The core step of the MVMD algorithm is to iteratively update mode $u_{k,c}\;(\omega )$, center frequency $\omega_{k}$, and the Lagrange multiplier λ. More specific details can be found in [23]. For the convenience of calculation, we have discretized and derived the iterative update formula as follows:

(1) Mode Update:

(22) $$u_{k,c}^{n+1}(\omega )=\frac{d_{c}(\omega )-\sum_{i=1,i\neq k}^{K}u_{i,c}^{n}(\omega )+\frac{\lambda_{c}^{\;n}(\omega )}{2}}{1+2\alpha {\left(\omega -\omega_{k}^{n}\right)}^{2}}$$

where *n* represents the number of iterations. ω represents frequency. $u_{k,c}\;(\omega )$ represents the *k*-th modal component under the *c*-th channel. $d_{c}(\omega )$ represents the frequency domain signal under the *c*-th channel. α represents the penalty coefficient.

(2) Center Frequency Update:

(23) $$\omega_{k}^{n+1}=\frac{\sum_{c=1}^{C}\sum_{i=0}^{N/2}\omega {\left|u_{k,c}^{n+1}(\omega_{i})\right|}^{2}}{\sum_{c=1}^{C}\sum_{i=0}^{N/2}{\left|u_{k,c}^{n+1}(\omega_{i})\right|}^{2}}$$

(3) Lagrange Multiplier Update:

(24) $$\lambda_{c}^{\;n+1}(\omega )=\lambda_{c}^{\;n}(\omega )+\tau \left(d_{c}(\omega )-\sum_{k=1}^{K}u_{k,c}^{n+1}(\omega )\right)$$

where τ represents the time constant.

After obtaining K modal components through the MVMD algorithm, calculate the correlation coefficient between modal components and the original signal as follows:

(25) $$r_{k,c}=\frac{\sum_{i=1}^{N}\left(u_{k,c}^{i}-{\bar{u}}_{k,c}\right)\left(d_{c}^{i}-{\bar{d}}_{c}\right)}{\sqrt{\sum_{i=1}^{N}{\left(u_{k,c}^{i}-{\bar{u}}_{k,c}\right)}^{2}}\sqrt{\sum_{i=1}^{N}{\left(d_{c}^{i}-{\bar{d}}_{c}\right)}^{2}}}$$

The modal component corresponding to the maximum correlation coefficient is expressed by $u_{best}$; (1) should be rewritten as follows:

(26) $$u_{best}=Hx+\varepsilon +b_{f}$$

### 2.4. Verify the Superiority of MVMD Signal Decomposition

(1) Modal alignment properties

Modal alignment of multivariate signals means that the signal components with the same frequency band of the mode can be decomposed into the mode at the same time in the same modal component. This is an extremely important property for multivariate data parallel analysis or information fusion. Especially in signal processing, the decomposed modal components need to be used for signal reconstruction. If there are oscillatory signals with different frequency bands in the same modal component, the extracted multivariate signal features will lose significance in practical engineering evaluation.

**Figure 5:**
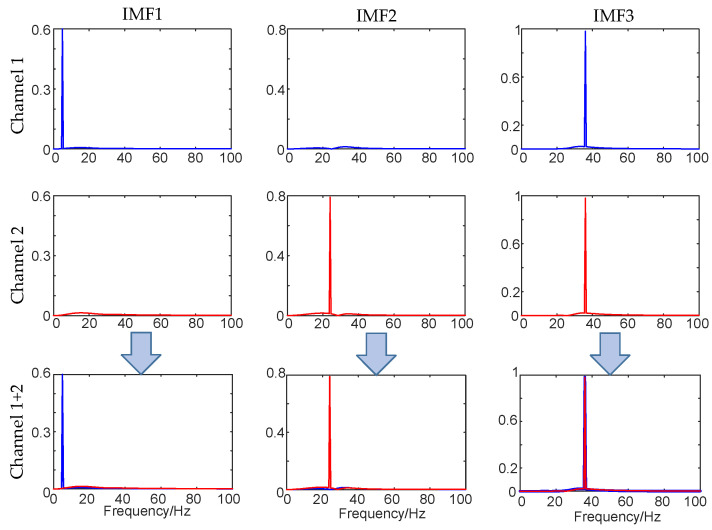
The decomposition results of MVMD for multivariate signals.

**Figure 6:**
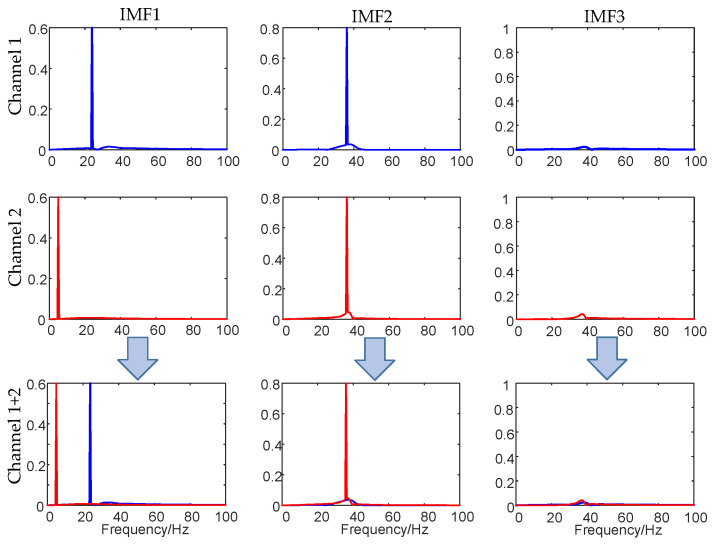
The decomposition results of VMD for multivariate signals.

Figure 5 and Figure 6 are the decomposition results of the simulation signal using MVMD and VMD, respectively. The simulation signal is a dual-channel signal. Channel 1 is composed of 5 Hz and 36 Hz signal components, and channel 2 is composed of 24 Hz and 36 Hz signal components. When the simulated signal is decomposed, the modal number *K* of the two algorithms is set to 3. It can be seen from Figure 5 that the 5 Hz signal component of channel 1 is decomposed into IMF1, IMF2 is the 24 Hz signal component of channel 2, and the common component, a 36 Hz signal of the two channels, is extracted into IMF3. In Figure 6, the 5 Hz signal component in channel 1 and the 24 Hz signal component in channel 2 are decomposed into the same IMF1, and modal aliasing occurs, which fully reflects the characteristics of modal alignment of the MVMD algorithm in processing multivariate signals and shows the superiority of MVMD over VMD in multivariate signal processing.

(2) Filtering characteristics of noise

**Figure 7:**
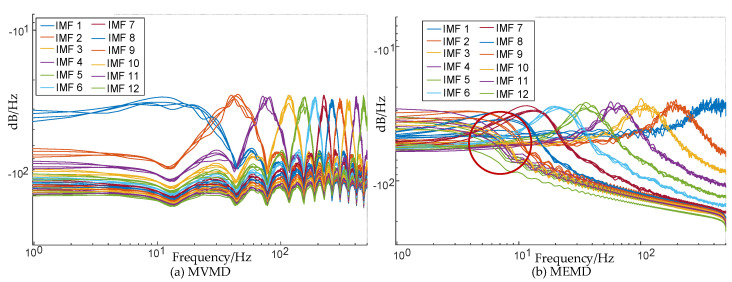
Comparison of filtering characteristics. (**a**) Filtering characteristics of decomposed signals using MVMD. (**b**) Filtering characteristics of decomposed signals using MEMD. The red circle indicates the occurrence of modal aliasing.

Both MVMD and multivariate empirical mode decomposition (MEMD) [29] have a parallel filtering structure similar to a wavelet filtering frame for Gaussian noise. To verify the filtering performance of these two algorithms, 50 Gaussian white noise samples with a signal-to-noise ratio of 20 dB and a channel number of 4 samples with a length of L = 1500 are set for decomposition. Figure 7 shows the decomposition results of the two algorithms. MEMD adaptively decomposes the Quaternary signal into 12 modal components, and sets the parameter *K* of MVMD to 12 for comparison. Comparing the two groups of curves, it can be observed that both algorithms have modal alignment characteristics, and the same frequency band components of multivariate signals are decomposed into the same mode, but for Gaussian white noise, MEMD presents a quasi-dual filter bank structure, with a wider frequency band and aliasing between frequency bands, as shown in the red circle in the figure. In contrast, the filtering structure of MVMD is better. There is a clear boundary between each modal component, which can orderly score the whole frequency domain of multivariate signals into different frequency bands, and the modal components in the same frequency band converge strictly.

### 2.5. Influence of Parameters on MVMD

**Figure 8:**
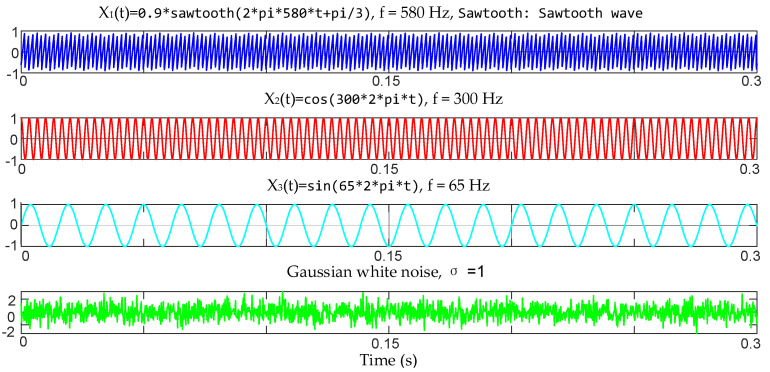
Simulated signal. *: multiplication sign.

To verify the specific impact of the setting of the key parameter, namely, the intrinsic mode number *K* and penalty parameter α, on the decomposition effect of MVMD, a simulation signal X(t) is set for inspection, as shown in Figure 8.

**Figure 9:**
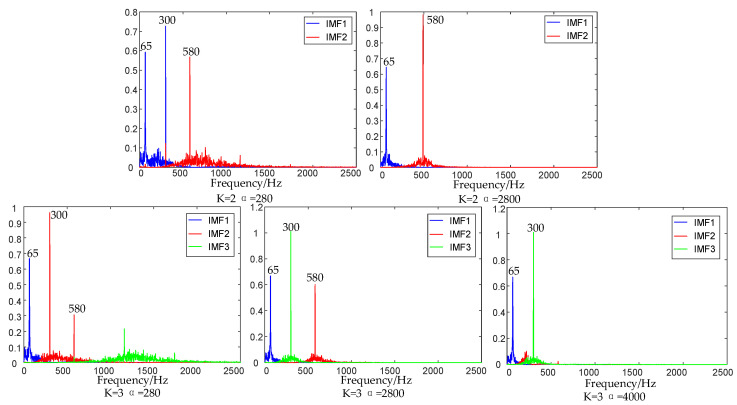
Effect of parameters on MVMD.

The influence of parameters on MVMD is shown in Figure 9. The intrinsic mode number K is set to 2 and 3, respectively, and the penalty parameter α is set to 280, 2800, and 4000, respectively, for analysis and comparison. When K= 2, the signal is under-decomposed, and the low-frequency 65 Hz signal and high-frequency 580 Hz signal are decomposed into one modal component. If α = 280, the bandwidth of each component becomes larger, and the problem of modal aliasing is prone to occur between the frequency domains of two adjacent modal components. When α = 2800, the frequency domain bandwidth of the modal component becomes smaller, and the 300 Hz IF signal is discarded in the decomposition process. When K = 3 and α = 280, the frequency bandwidth between the modal components decomposed by MVMD is wide. IMF2 contains two components, 300 Hz and 580 Hz, and modal aliasing occurs; when K = 3 and α = 2800, there is a clear boundary between the various modal components, which has a good decomposition effect. When K = 3 and α = 4000, the signal component frequency band is narrow and the high-frequency signal is lost. To sum up, the parameter K mainly affects the number of modal components of the final decomposition of the signal, and the penalty parameter α mainly affects the convergence of the central frequency band of the modal components. When the value is small, the adjacent modes are prone to overlapping. When the value is too large, the convergence of the signal to the central frequency is too large, which will lead to the rejection of the high-frequency modal components.

In a word, the selection of appropriate parameters K and α is crucial and has a great impact on the decomposition effect of MVMD and subsequent feature extraction, and the optimization process of these two parameters is very important.

### 2.6. Parameter Optimization of MVMD Based on Multi-Index Fitness Function WOA

MVMD has certain advantages in parallel processing and feature extraction of multivariate signals, but the main parameters are set according to human experience, and the effect of modal decomposition is greatly affected by human subjectivity, so the algorithm itself cannot realize adaptive decomposition according to the essential characteristics of the original signal. The WOA can solve this problem well.

Compared with other heuristic optimization algorithms, WOA adopts two behaviors of shrinking and surrounding and spiral updating position at the same time in the stage of bubble network attack. This dual mechanism gives it a strong fine search ability in local development and has fewer mathematical model parameters. The search step is dynamically adjusted by the convergence factor that varies with the number of iterations. At the beginning of the iteration, it tends to explore globally in a wide range to avoid prematurity, and at the end of the iteration, it gradually turns to local development. Because it only depends on the input and output of the objective function, there is no requirement for the continuity, differentiability, or convexity of the function. Generally, the acceptable, approximately optimal solution can be found in a few iterations. The core mechanism is mainly divided into three stages: surrounding prey, bubble net attack, and searching for prey.

(1) Surrounding prey:

Humpback whales can identify and encircle the general location of prey, but the exact position of the prey remains unclear within this vast space. Therefore, each whale must rely on continuous interaction, communication, and information sharing, as well as random movement, to detect and locate prey. In the WOA, this phase assumes the current optimal whale’s position as the food location, with other individuals moving to surround and prey on the food. The mathematical model is as follows:

(27) $$\left\{\begin{array}{l} X(t+1)=X*(t)-AD\\ D=\left|CX*(t)-X(t)\right| \end{array}\right.$$

where X(t) represents the position of the whale. X*(t) represents the current optimal position. A and C represent coefficient vectors. D represents the current distance between the whale and its prey. t represents the number of iterations.

(2) Bubble net attack:

Humpback whales use a bubble net to approach their prey in a spiral shape, forcing them into a predicament. Bubble net attack is a hunting process in which whales spiral upwards and contract to encircle. WOA uses a logarithmic spiral to simulate the spiral enclosure of whales as follows:

(28) $$\left\{\begin{array}{l} X(t+1)=D\prime e^{bl}\cos(2\pi l)+X*(t)\\ D\prime =\left|X*(t)-X(t)\right| \end{array}\right.$$

where b represents a constant that defines the shape of a spiral. l represents a constant in [−1, 1]. D′ represents the optimal distance obtained so far. During bubble net hunting, humpback whales surround their prey along a spiral path. To obtain a model of simultaneous behavior, assuming a 50% probability of choosing between contraction, encirclement predation, or spiral encirclement, the mathematical model is as follows:

(29) $$X(t+1)=\left\{\begin{array}{l} X*(t)-AD,p<0.5\\ D\prime e^{bl}\cos(2\pi l)+X*(t),p\geq 0.5 \end{array}\right.$$

where p represents a random number within the range of [0, 1].

(3) Search for prey:

In addition to the bubble net predation strategy, individual whales will carry out a random search through dynamic adjustment A during the search process. When the absolute value of A exceeds 1, the algorithm will randomly select other individual positions as reference points to adjust the search direction, which effectively enhances the algorithm’s ability to break through local optima. The mathematical model for random search is as follows:

(30) $$\left\{\begin{array}{l} X(t+1)=X_{rand}(t)-AD\\ D=\left|CX_{rand}(t)-X(t)\right| \end{array}\right.$$

where $X_{rand}(t)$ represents a randomly selected whale.

In order to solve the influence of the parameter setting of MVMD on the decomposition results, a multi-index of minimum envelope entropy, spectral compactness, and correlation coefficient is proposed based on WOA, which is used as the fitness function to seek the optimal solution of (K,α). The multivariate signals are decomposed according to the optimal value, and the characteristic signals are extracted.

(1) Envelope entropy calculation:

(31) $$\left\{\begin{array}{l} \bar{E}=-\frac{1}{K}\sum_{k=1}^{K}\sum_{t=1}^{N}p_{k}(t){\;\log}_{2}\left(p_{k}(t)\right)\\ p_{k}(t)=\frac{a_{k}(t)}{\sum_{t=1}^{N}a_{k}(t)}\\ a_{k}(t)=\sqrt{\sum_{c=1}^{C}{\left|H\left\{u_{k,c}(t)\right\}\right|}^{2}} \end{array}\right.$$

where $a_{k}(t)$ represents the envelope signal. $p_{k}(t)$ represents the normalized envelope signal. N represents the signal length. $\bar{E}(t)$ represents envelope entropy. H represents the Hilbert transform. K represents the number of modal decompositions.

(2) Correlation coefficient calculation:

(32) $$\left\{\begin{array}{l} \bar{\rho }=\frac{1}{K}\sum_{k=1}^{K}\frac{1}{C}\sum_{c=1}^{C}\left|\rho_{ij,k,c}\;\right|\\ \rho_{ij,k,c}\;=\frac{\mathrm{Cov}(u_{i,k,c},u_{j,k,c})}{\sigma_{u_{i,k,c}}\sigma_{u_{j,k,c}}} \end{array}\right.$$

where $\mathrm{Cov}(u_{i,k,c},u_{j,k,c})$ represents covariance.

(3) Spectral compactness:

(33) $$\left\{\begin{array}{l} \bar{S}=\frac{1}{K}\sum_{k=1}^{K}\frac{1}{C}\sum_{c=1}^{C}S_{k,c}\\ S_{k,c}=\sum_{\omega =0}^{\omega /2}{(\omega -\omega_{k,c})\;}^{2}P_{k,c}^{\prime }\;(\omega )\\ P_{k,c}^{\prime }\;(\omega )=\frac{P_{k,c}\;(\omega )}{\sum_{\omega =0}^{\omega /2}P_{k,c}\;(\omega )} \end{array}\right.$$

where $\bar{S}$ represents spectral compactness. $P_{k,c}\;(\omega )$ represents power spectral density.

(4) Comprehensive fitness value:

(34) $$C_{FV}=w_{1}{\bar{E}}_{k}+w_{2}{\bar{\rho }}_{i,j,c}+w_{3}\bar{S}$$

where $w_{1}$, $w_{2}$ and $w_{3}$ represent weights.

**Figure 10:**
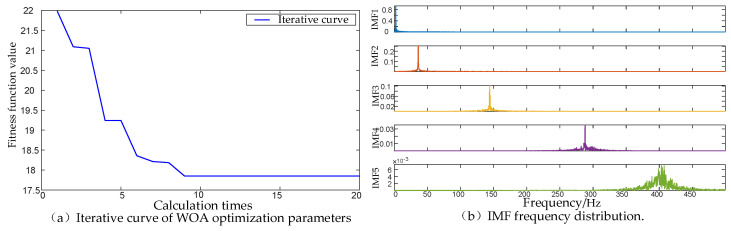
Simulation results of the proposed method. (**a**) The process of fitness function value changing with the number of iterations when using WOA to optimize parameters. (**b**) The IMF frequency distribution of MVMD when using optimal parameters.

We simulated a 3-channel signal to verify the effectiveness of the proposed method. The frequency components of the simulated signal are shown in Table 1 below, and the simulation results are shown in Figure 10. As can be seen from Figure 10a, the optimization process of the proposed method converges quickly without falling into the local optimal solution. Figure 10b shows that the MVMD under the optimal parameters correctly decomposes the four main frequency components in the multivariate signals, which proves that the proposed method is effective and has excellent performance.

### 2.7. The Overall Structure of WOA-MVMD-GLT

**Figure 11:**
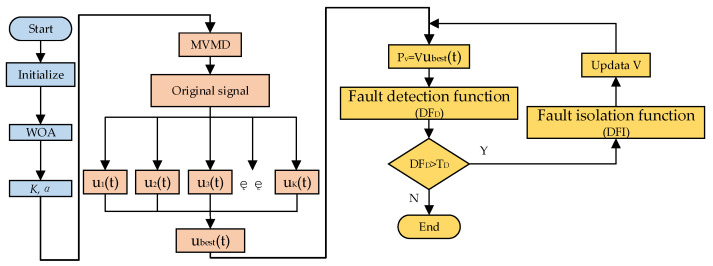
The flowchart of WOA-MVMD-GLT.

In this paper, the flowchart of WOA-MVMD-GLT is shown in Figure 11.

(1) Initialize the WOA parameters.
(2) Using WOA parameter optimization, the best combination parameter (K, α) is obtained.
(3) The collected original data is calculated by the MVMD algorithm to output K characteristic modes.
(4) The correlation coefficient between each modal component and the original signal is calculated, and the characteristic mode corresponding to the maximum correlation coefficient is represented by $u_{best}$.
(5) The parity vector is calculated to construct the function of fault detection and fault isolation.
(6) If a fault is detected, the fault is immediately isolated, and the matrix V is updated after isolating the faulty sensor. The specific steps for updating the parity matrix are as follows: after isolating the fault sensor, the corresponding column vector of the fault sensor in the parity matrix V is deleted, the parity matrix V is reconstructed, and the previous matrix is replaced for the next round of fault detection and isolation.

## 3. Experiment

**Figure 12:**
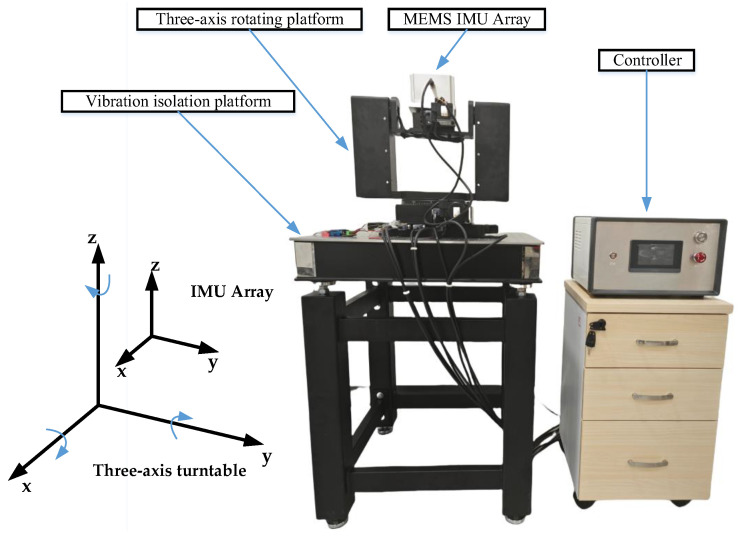
The experimental setup.

The developed MEMS IMU array prototype is employed to validate the effectiveness of the proposed method for fault detection and isolation. We placed the MEMS IMU array on a three-axis rotating table and simulated its motion based on a preset trajectory, and the output data of the MEMS IMU array was collected by a computer through RS-422 communication. The acquisition device is a serial port acquisition card. The x, y, and z axes of the three-axis turntable are set to 10°/s and rotate at the same time. The repeatability accuracy of the rotating device is ± 0.05°, the absolute accuracy is ± 0.06°, the resolution is 0.025°, and the parallelism is 15 μm. The experimental setup is shown in Figure 12, and the MEMS IMU used is shown in Table 2.

**Figure 13:**
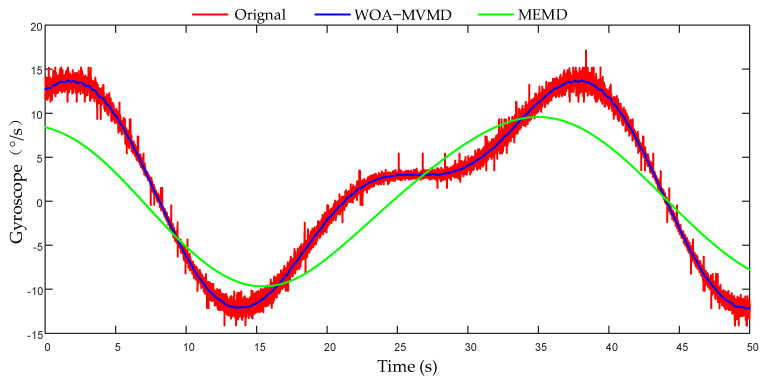
Comparison of signal noise reduction.

Take the gyroscope as an example; the comparison between the characteristic mode with signal characteristics after noise reduction and the original signal is shown in Figure 13. The effects of some sensors are shown in Table 3. It can be seen that when using WOA-MVMD, gyroscopes and accelerometers can effectively extract signal features from the original signal containing noise in a dynamic environment. However, when using the MEMD method, the required characteristic signals cannot be extracted completely, which is caused by the phenomenon of modal aliasing, which is consistent with the above simulation results. The signal-to-noise ratio of the extracted feature signal is significantly improved, indicating that the noise has been significantly suppressed.

**Figure 14:**
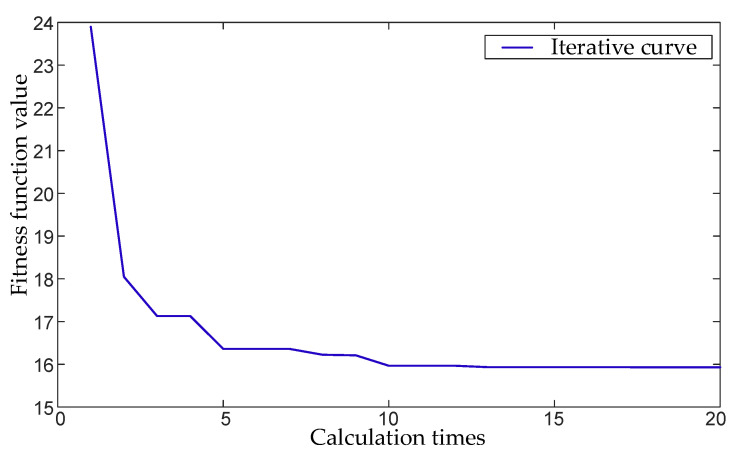
WOA parameter optimization iteration curve of actual data.

**Figure 15:**
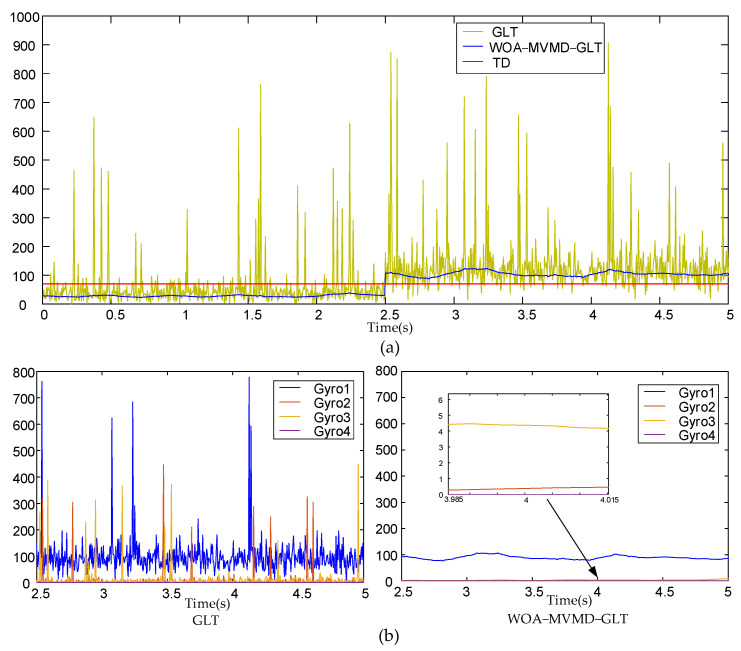
(**a**) Fault detection curve. (**b**) Comparison of fault isolation curves. The curve of gyro 4 almost coincides with the coordinate axis. There are many sensors, but only some are listed.

We inject a 1°/s step fault into the gyroscope 1 of the inertial measurement array at 2.5 s and collect data to verify the effectiveness of the proposed method. The iteration curve of the WOA parameter optimization of the actual data is shown in Figure 14. It can be seen that the iteration process is fast and there is no local optimization. The fault detection and fault isolation curve is shown in Figure 15. As can be seen from Figure 15a, due to the interference of abnormal points and noise, many faults were detected when only the GLT method was used before 2.5 s, and the false alarm rate was high. After 2.5 s, the false alarm rate was also high. The proposed method can effectively suppress the problem of low fault detection rate caused by interference. Figure 15b shows that this method significantly improves the accuracy of fault isolation. Quantitative statistical analysis of false alarm rate, missed alarm rate, and false isolation rate with a fault amplitude of 0.9°/s are shown in Table 4.

Drift faults are a common type of incipient failure in IMUs, which can be caused by adverse environmental impact, internal wear, and sensor aging. When a drift fault occurs, the measured output value usually deviates from the normal value at a specific rate. The mathematical model is as follows:

(35) $$o{\left(t\right)}_{f}=o(t)+k(t-t_{f}),t\geq t_{f}$$

where $o{\left(t\right)}_{f}$ represents the measurement output in case of failure. o(t) the normal measurement output. k represents the drift coefficient. $t_{f}$ represents the time when the fault occurs.

At 2.5 s, we added an early fault with a drift coefficient of 0.1 to gyro 1. The delay time of fault diagnosis in the GLT method is 0.04 s, and the delay time of fault diagnosis in the proposed method is 0.03 s, which is relatively reduced by 25%, significantly improving the real-time performance of diagnosis.

## 4. Conclusions

In this paper, a new method for WOA-MVMD-GLT is proposed. Aiming at the influence of interference on FDI of MEMS IMU array, the feature extraction algorithm is combined with the traditional parity vector method to detect and isolate the fault of the MEMS IMU array in dynamic environments. Experiments show that the proposed method can improve the accuracy of the fault detection and isolation, and the performance is significantly better than the traditional method.

The experimental results show that this method can solve the interference problem of sensor output to a certain extent. The fault detection and isolation method of MVMD-GLT provided a new idea for the FDI of the MEMS IMU array, but there are still some shortcomings that require further exploration, summarized as follows:

(1) With the rapid development of intelligent algorithms, the use of intelligence for further research;
(2) At present, the research is not deep enough. We hope to find a better method to continue optimization in the future.

## Figures and Tables

**Table 1:** Frequency value of each channel signal.

	Channel 1	Channel 2	Channel 3
Frequency	2 Hz	2 Hz	2 Hz
	144 Hz	36 Hz	36 Hz
	288 Hz	288 Hz	144 Hz
Noise	σ=0.1	σ=0.08	σ=0.12

**Table 2:** The MEMS IMU used in the experiment.

Type	Manufacturer	Number
MPU-6500	InvenSense Inc., San Jose, CA, USA	16

**Table 3:** Comparison of signal-to-noise ratio.

Type	SNR: Original Signal	SNR: After Noise Reduction	Type	SNR: Original Signal	SNR: After Noise Reduction
Gyro 1	−3.013	19.715	Acce 1	−5.423	12.581
Gyro 2	3.221	41.024	Acce 2	6.713	37.147
Gyro 3	−3.371	32.051	Acce 3	9.43	41.313
Gyro 4	−3.013	16.171	Acce 4	−5.123	4.457
Gyro 5	3.406	41.157	Acce 5	6.494	38.007
Gyro 6	−2.917	31.782	Acce 6	11.134	40.822
Gyro 7	−4.184	17.711	Acce 7	−5.846	5.725

**Table 4:** Quantitative analysis of algorithm performance.

Method	False Alarm Rate	Missed Alarm Rate	Comprehensive Accuracy of Fault Detection	False Isolation Rate	Accuracy of Fault Isolation
GLT	4.98%	26.58%	68.44%	4.2%	95.8%
Proposed	0%	1.5%	98.5%	0%	100%

## Data Availability

The original contributions presented in this study are included in the article. Further inquiries can be directed to the corresponding author.
